# Concurrent Effects of Sediment Accretion and Nutrient Availability on the Clonal Growth Strategy of *Carex brevicuspis*-A Wetland Sedge That Produces Both Spreading and Clumping Ramets

**DOI:** 10.3389/fpls.2017.01685

**Published:** 2017-09-27

**Authors:** Xinsheng Chen, Yulin Liao, Yonghong Xie, Feng Li, Zhengmiao Deng, Zhiyong Hou, Chao Wu

**Affiliations:** ^1^Key Laboratory of Agro-ecological Processes in Subtropical Region, Institute of Subtropical Agriculture, The Chinese Academy of Sciences, Changsha, China; ^2^Dongting Lake Station for Wetland Ecosystem Research, Institute of Subtropical Agriculture, The Chinese Academy of Sciences, Changsha, China; ^3^Soil and Fertilizer Institute of Hunan Province, Hunan Academy of Agricultural Sciences, Changsha, China

**Keywords:** clonal plant, growth form, environmental stress, plant response, clonal architecture

## Abstract

Clonal plants producing both clumping and spreading ramets can adjust their growth forms in response to resource heterogeneity or environmental stress. They might produce clumping ramets to retain favorable patches, or produce spreading ramets to escape from stress-affected patches. This study aimed to investigate the rarely reported concurrent effects of sediment accretion and nutrient enrichment, which often occur simultaneously in lacustrine wetlands, on the vegetative propagation and clonal growth forms of *Carex brevicuspis* C.B. Clarke by conducting a factorial experiment of sediment burial and nutrient addition. Biomass accumulation, new ramet and rhizome numbers, and ramet length of *C. brevicuspis* were not affected at moderate burial, but were significantly lower after deep burial. Similarly, nutrient enrichment increased the growth and vegetative propagation of *C. brevicuspis* up to moderate sediment burial, but not after deep burial. Sediment accretion increased the proportion of spreading ramets produced by *C. brevicuspis*, whereas nutrient addition had no effect on the clonal growth forms. Our results indicated that the plasticity of clonal growth forms is an effective strategy used by plants to acclimate to moderate sediment accretion. Nutrient enrichment did not influence the clonal growth forms of *C. brevicuspis* and could not facilitate its acclimation to heavy sedimentation condition.

## Introduction

Clonal plants dominate several widespread ecosystems such as many grasslands and wetlands, where both biotic and abiotic factors are heterogeneous in space and time (Klimeš et al., 1997; Gough et al., 2002). They have developed various growth strategies to cope with environmental heterogeneity such as resource availability and biotic competition (Schmid and Harper, 1985; Cheplick, 1997; Puijalon et al., 2007). Their growth strategies can be categorized as phalanx type (constituting a compact structure of loosely spaced ramets) and guerrilla type (spreading ramets forming a loosely arranged group of widely spaced ramets), which represent the endpoints in a continuum of possible growth forms (Lovett-Doust, 1981; Bernard, 1990; de Kroon and Hutchings, 1995). These two growth strategies might have special importance to the ecology and evolution of clonal plants (Bernard, 1990; Cheplick, 1997). The phalanx strategy might enable clonal plants to retain favorable patches, facilitating better use of locally abundant resources at a favorable microsite (Bernard, 1990; de Kroon and Hutchings, 1995; Ye et al., 2006). In contrast, the guerrilla growth form enables plants to escape from less favorable patches where resource levels are low or competitive stress is high (Humphrey and Pyke, 2001; Amiaud et al., 2008; Chen et al., 2011; Li et al., 2015).

A majority of clonal plant species use either a phalanx-like or guerrilla-like growth strategy (Lovett-Doust, 1981; Humphrey and Pyke, 1998). However, many sedge species and some grass species produce both clumping and spreading ramets, resulting in a combined growth form (Bernard, 1990; Ye et al., 2006; Amiaud et al., 2008; Martina and von Ende, 2013). Previous studies indicated that such plant species can adjust their clonal growth forms in response to resource availability or sediment accretion (Ye et al., 2006; Chen et al., 2011). With higher nutrient supply, *Leymus secalinus* (Georgi) Tzvel. produced fewer spreading ramets and a greater number of clumping ramets (Ye et al., 2006). With increasing sediment burial depth, *Carex brevicuspis* remarkably decreased the proportion of clumping ramets, whereas markedly increased the proportion of spreading ramets (Chen et al., 2011). Therefore, nutrient enrichment and sediment accretion likely influence the clonal growth forms in an opposite manner.

Sediment accretion and nutrient enrichment often occur simultaneously in lacustrine wetlands (Maun, 1998; Frosini et al., 2012; Chen et al., 2017). Sediments induce hypoxia in the root zone and cause physical overburden to the apical meristems of buried plants (Chen et al., 2014a; Pan et al., 2014). Wetland macrophytes respond to burial by elongating stem, increasing stem biomass, and releasing dormant buds on their shoots and rhizomes (Maun, 1998; Pan et al., 2012; Chen et al., 2014a). Nutrient enrichment might facilitate the acclimation of macrophytes to sedimentation by increasing the shoot length, total biomass, and ramet numbers of wetland macrophytes (Xie et al., 2013; Chen et al., 2017). However, the two co-occurring factors might affect plant growth independently or by interacting such that one factor reduces or amplifies the impact of the other factor (Lenssen et al., 2003; Puijalon et al., 2007). For example, when plants were simultaneously subjected to waterlogged soils and canopy shade, waterlogging and shade independently affected the growth of the waterlogging-tolerant species, whereas waterlogging reduced the negative effect of shade on intolerant species (Lenssen et al., 2003). The combined effects of sediment accretion and nutrient availability on the clonal growth strategy of wetland plants have rarely been investigated.

In the present study, we experimentally investigated the concurrent effects of nutrient availability and sediment accretion on the plasticity of clonal growth forms in a rhizomatous sedge *C. brevicuspis*, one of the dominant macrophytes in Dongting Lake wetlands (Xie and Chen, 2008). It produces both spreading and clumping ramets, resulting in a combined growth form (Chen et al., 2011, 2014b). In particular, we tested the following hypotheses: (1) sediment accretion will decrease the growth and propagation of *C. brevicuspis*, whereas nutrient enrichment will alleviate the negative effect of sediment burial; and (2) sediment accretion will increase the proportion of spreading ramets produced by *C. brevicuspis*, whereas nutrient enrichment will increase the proportion of clumping ramets.

## Materials and Methods

### Study Species

*Carex brevicuspis* (Cyperaceae) is a perennial rhizomatous sedge distributed in eastern mainland China and Taiwan (Dai et al., 2010). The pseudoculm of the plant, consisting of a series of overlapping leaf sheaths, is usually 20–55 cm in height. *C. brevicuspis* plants flower and fruit from April to May in the Dongting Lake wetlands. However, seedlings are scarce in the field because plants recruit mainly by producing vegetative ramets from rhizomes (Chen et al., 2015, 2016).

### Experimental Design

The experiment was conducted on wild plants collected from a monitoring plot (112°47′11.6″E, 29°29′14.3″N) of the Dongting Lake Station for Wetland Ecosystem Research, the Chinese Academy of Sciences, Yueyang, Hunan Province, China. In an area of approximately 5 m^2^, plant fragments with rhizomes were dug up and transported to the Dongting Lake Station for Wetland Ecosystem Research on May 17, 2015. Plant fragments were planted at a depth of approximately 5 cm in a nursery bed containing a soil/sand mixture (1:1 v/v). The soil was collected from the upper layer of the plant collection site, which contained 1.02 mg total nitrogen and 0.38 mg total phosphorus per kg dry soil. After the establishment of plants from fragments, on June 17, 80 similar-sized plants were selected, planted into individual plastic containers (height, 27 cm; diameter, 24 cm) that were filled with 10 cm soil, and allowed to grow. On June 30, 48 containers with similar-sized plants (5–7 leaves and 22–25 cm in height) were selected for the experiment.

The experimental design was a randomized block with eight replicates; the experiment was performed in separate outdoor water tanks (200 cm × 200 cm × 100 cm) containing water at a depth of 10 cm. Three levels of sediment accretion (0, 5, and 10 cm) and two levels of nutrient (no addition and nutrient addition) were used, with each combination applied to one of the six plants in each tank. Sediment can accrete 3–7 cm annually in Dongting Lake wetlands; therefore, 10 and 5 cm were chosen as high and moderate sedimentation, respectively (Xie and Chen, 2008; Li et al., 2016). During the experiment, two types of sediments were prepared. Sand collected from Dongting Lake, which contained 0.02 mg total nitrogen and 0.04 mg total phosphorous per kg dry sand, was used as the no-addition sediment. Nutrient-addition sediment was obtained by homogeneously mixing sand with slow-release fertilizer (1.5 g slow-release fertilizer per kg sand). The slow-release fertilizer was 312S Osmocote Exact (N-P-K, 15-9-11 + 2MgO; The Scotts Company, United States). Approximately 2.5 or 5.0 kg of sediment (corresponding to a layer of sediment approximately 5 or 10 cm deep), with or without nutrient addition, was added to each container for the 5- or 10 cm burial treatment. For treatment at 0 cm burial with nutrient addition, 1.5 g slow-release fertilizer was applied to the soil surface and covered with a thin layer of sand (less than 0.2 cm). A thin layer of sand was also added for the treatment at 0 cm burial without nutrient addition.

The water level in the tanks was maintained at 10 cm (0 cm for the plants), with tap water added as required and surplus water removed after rain. The position of containers in each tank was changed weekly to reduce the effect of light heterogeneity. Plants were checked each week, and new ramets were marked with plastic tags. Meteorological data were recorded using an automatic weather station (Milos 520; Vaisala, Finland) located approximately 150 m away from the experimental site. During the experiment (June 30–October 24, 2015), daily air temperature was 25.4 ± 3.3°C (mean ± SD).

### Harvest and Measurement

The plants were harvested 117 days after treatment. Plants with any live aboveground material were defined as alive. The plants were carefully excavated from the tanks to maintain the connections between ramets, cleaned with tap water, and transported to the laboratory for measurements. The number of ramets and rhizomes and the length of new ramets and spacers produced by each plant were recorded. The length of ramets was calculated as the distance from the shoot base to the tip of the longest leaf. The length of spacers was defined as the distance of rhizomes from each ramet to the original plant (Chen et al., 2011). Each plant was then separated into shoots, roots, and rhizomes. The biomass of each plant part was measured after drying at 80°C for 48 h in an oven. Biomass accumulation was calculated as the total plant dry weight at the end of the experiment. Clumping ramets were defined as ramets produced from shortened rhizomes (spacer length usually less than 1 cm), whereas spreading ramets were those produced from the distal part of long rhizomes (Chen et al., 2011).

### Data Analysis

The significance of differences in biomass accumulation, the number of new ramets and rhizomes, the length of ramets and spacers, and the proportion of spreading ramets under different sedimentation depth and nutrient levels was assessed using general linear models, with sedimentation depth and nutrient level included as main factors and block as a random factor (McKone and Lively, 1993). Multiple comparisons of means were performed using Tukey’s test at the 0.05 significance level; Bonferroni corrections for multiple comparisons were applied as appropriate. Data were log^10^-transformed if necessary to reduce the heterogeneity of variances, and homogeneity was confirmed using Levene’s test. All statistical analyses were performed using SPSS 15.0 (SPSS Inc., United States).

## Results

### Biomass Accumulation and Allocation

**FIGURE 1:**
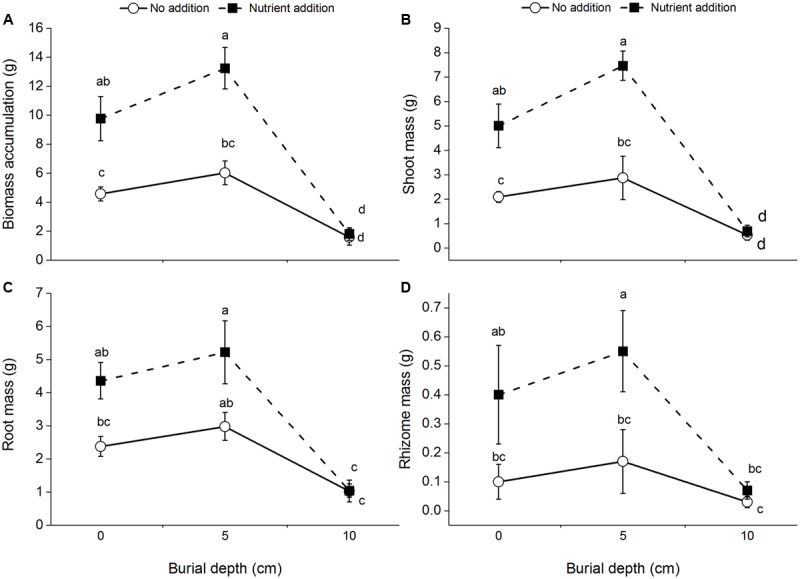
Biomass accumulation **(A)**, shoot mass **(B)**, root mass **(C)**, and rhizome mass **(D)** of *Carex brevicuspis* growing at three sedimentation depths with two nutrient levles. Standard error bars sharing the same lowercase letters are not significantly different (*P* > 0.05).

Regardless of burial depth and nutrient level, all the plants survived. Biomass accumulation and shoot mass of *C. brevicuspis* were significantly affected by burial depth and nutrient level, with significant interactions between both the factors (**Table 1** and **Figures 1A,B**). Further, they were lower at 10 cm burial depth than at 0 and 5 cm burial depths. Nutrient addition increased the total biomass and shoot mass, except at 10 cm burial depth (**Figures 1A,B**).

**Table 1:** Summary of three-way analysis of variance (*F*-values) for shoot, root, rhizome mass, and total biomass; ramet and spacer length; ramet and rhizome numbers; and the proportion of spreading ramets in *Carex brevicuspis* growing at three sedimentation depths with two nutrient levels.

Effect	Biomass accumulation	Shoot mass	Root mass	Rhizome mass	Ramet length	Spacer length	Number of ramets	Number of rhizomes	Proportion of guerrilla ramets
Burial depth (B)	62.36^∗∗∗^	86.24^∗∗∗^	28.46^∗∗∗^	7.68^∗∗^	21.45^∗∗∗^	2.55^ns^	32.68^∗∗∗^	8.24^∗∗^	8.51^∗∗^
Nutrient level (N)	30.93^∗∗∗^	43.92^∗∗∗^	10.84^∗∗^	15.13^∗∗∗^	2.69^ns^	1.7^ns^	21.27^∗∗∗^	7.58^∗∗^	0.16^ns^
Block	2.26^ns^	3.02^∗^	1.21^ns^	2.06^ns^	1.14^ns^	2.03^ns^	2.00^ns^	0.74^ns^	0.86^ns^
B × N	5.50^∗∗^	8.01^∗∗^	1.95^ns^	1.68^ns^	0.12^ns^	0.01^ns^	0.59^ns^	0.53^ns^	1.04^ns^


^∗∗∗^*P* < 0.001, ^∗∗^*P* < 0.01, ^∗^*P* < 0.05, ^ns^*P* > 0.05.

Root mass and rhizome mass of *C. brevicuspis* were significantly affected by burial depth and nutrient level (**Table 1** and **Figures 1C,D**). Without nutrient addition, root mass at the 10 cm burial depth was lower than that at 0 and 5 cm burial depths. With nutrient addition, root mass and rhizome mass were lower at 10 cm burial depth than at 5 cm burial depth.

### Length of Ramets and Spacers

**FIGURE 2:**
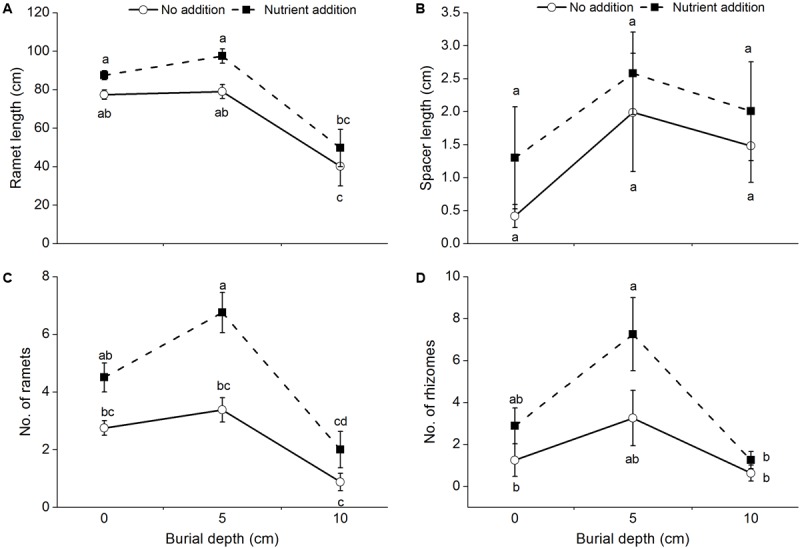
The length of ramets **(A)**, spacers **(B)**, the number of ramets **(C)**, and rhizomes **(D)** of *C. brevicuspis* growing at three sedimentation depths with two nutrient levels. Standard error bars sharing the same lowercase letters are not significantly different (*P* > 0.05).

The length of ramets produced by *C. brevicuspis* was significantly affected by burial depth (**Table 1** and **Figure 2A**) and was lower at 10 cm burial depth than at 0 and 5 cm burial depths. The length of spacers produced by *C. brevicuspis* ranged from 0.42 to 2.01 cm, but was not significantly affected by burial depth and nutrient level (**Table 1** and **Figure 2B**).

### Number of New Ramets and Rhizomes

The number of ramets and rhizomes produced by *C. brevicuspis* was significantly affected by burial depth and nutrient level (**Table 1** and **Figures 2C,D**). The number of ramets at 10 cm burial depth was lower than that at 0 and 5 cm burial depths (**Table 1** and **Figure 2C**). Their number increased with nutrient addition at 5 cm burial depth (**Figure 2C**) and was higher than that at 10 cm burial depth (**Figure 2D**).

### The Proportion of Spreading Ramets to Total Ramets

**FIGURE 3:**
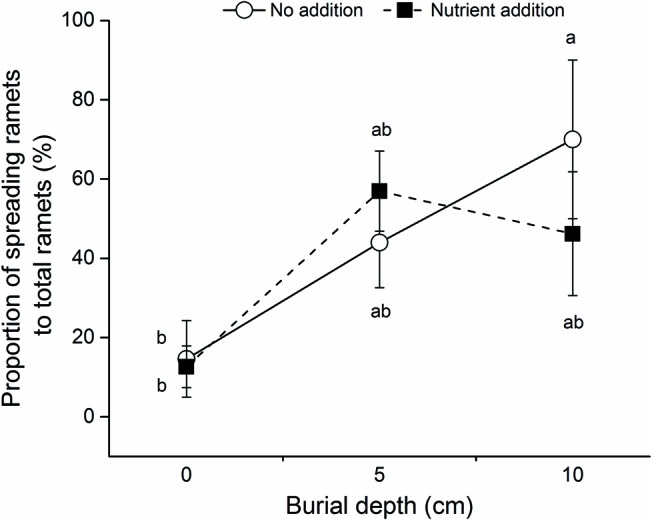
Proportion of spreading ramets to total ramets of *C. brevicuspis* growing at three sedimentation depths with two nutrient levels. Standard error bars sharing the same lowercase letters are not significantly different (*P* > 0.05).

The proportions of spreading to total ramets produced by *C. brevicuspis* were significantly affected by burial depth, but not by nutrient level (**Table 1** and **Figure 3**). Without nutrient addition, the proportion of spreading ramets at 10 cm burial depth was higher than that at 0 cm burial depth (**Figure 3**).

## Discussion

We intended to identify the respective influences of sediment accretion and nutrient availability on the clonal growth strategies of *C. brevicuspis*. Biomass accumulation, number of new ramets and rhizomes, and length of ramets were not reduced at moderate burial, but were decreased significantly at deep burial. This suggested that *C. brevicuspis* can acclimate to moderate burial, but not to deep burial. Morphologically, *C. brevicuspis*, a non-stem sedge, escapes from sediment burial by producing new spreading ramets through rhizome growth and elongation (Chen et al., 2011). Physiologically, it can acclimate to sedimentation-induced anoxia via carbohydrate metabolism (Pan et al., 2012). The process of projecting new ramets above the sediment surface might require more energy resources than that needed for vertical shoot elongation, as in many caulescent grasses (Maun, 1998; Deng et al., 2008; Chen et al., 2014a). Therefore, *C. brevicuspis* could acclimate to moderate sedimentation, but not to deep sedimentation like some caulescent macrophytes when the energy storage is limited. Our results are consistent with the field distribution of *C. brevicuspis* in Dongting Lake wetlands: *C. brevicuspis* grows in mid-elevation sites where sedimentation rate is moderate (Li et al., 2016).

Nutrient supply increased biomass accumulation and vegetative propagation of *C. brevicuspis* up to moderate burial, but had no effect after deep burial. This result only partially supported our first hypothesis. Nutrient input generally increased biomass production, increased allocation to shoots, and increased the rates of clonal spread and tiller production (Wetzel and van der Valk, 1998; Maurer and Zedler, 2002; Xie et al., 2013). However, during heavy sedimentation, especially when most of the photosynthetic tissues were buried, the energy needed during emerging process might mainly depend on carbohydrate reserves in the rhizomes (Deng et al., 2013; Chen et al., 2014a). Absorption and utilization of soil nutrients by *C. brevicuspis* were remarkably restricted by heavy sedimentation; therefore, sediment nutrients have no effect on the growth and propagation of *C. brevicuspis* under heavy sedimentation condition.

Sediment accretion increased the proportion of spreading ramets of *C. brevicuspis*, whereas nutrient level had no significant effect. This result only partially supported our second hypothesis. Our result that nutrients have no effect on the clonal growth forms of *C. brevicuspis* was inconsistent with those of previous studies on *L. secalinus*, which showed that nutrient supply increased the proportion of clumping ramets (Ye et al., 2006, 2015). Different responses of the two species to nutrient availability might be attributed to their specific clonal growth form. In general, *L. secalinus* is a guerrilla-like species because spreading ramets were dominant (over 50%) under various nutrient and light conditions (Ye et al., 2006, 2015). In this case, an increase in the proportion of clumping ramets might facilitate the utilization of locally abundant resources or consolidate patches (de Kroon and Hutchings, 1995; Ye et al., 2006). However, *C. brevicuspis* is a phalanx-like species under normal conditions (without sediment burial), because it primarily produces clumping ramets (over 80%; Chen et al., 2011, 2014b and references therein). In this case, nutrient addition might not further increase the proportion of clumping ramets of *C. brevicuspis*. Moreover, a certain proportion of spreading ramets might alleviate the intra-population competition in this phalanx-like species (Li et al., 2015). Based on the findings of previous studies and references therein (Ye et al., 2006, 2015), we inferred that spreading ramets might respond more sensitively to resources than clumping ramets.

According to the foraging theory of clonal plants, the function of clumping ramets is for consolidation, whereas that of spreading ramets is for expansion or escaping (Sutherland and Stillman, 1988; de Kroon and Hutchings, 1995; Ikegami et al., 2007). In response to single environmental factors such as nutrient availability or sediment accretion, a trade-off between clumping ramets (use of favorable patches) and spreading ramets (escape from stressful sites) might exist (Ye et al., 2006; Chen et al., 2011). However, the interactive effects of sediment accretion and nutrient availability on morphological plasticity might act in a hierarchical manner (Lenssen et al., 2003; Pan et al., 2014). Sedimentation is generally more inhibiting to wetland plant growth than nutrient availability owing to induced hypoxia in the root zone and physical stress over apical meristems (Maun, 1998). Plant morphology is likely to adjust to the primary limiting factor when different environmental factors occur simultaneously (Lenssen et al., 2003). Further investigation should be conducted to clarify the interactive effects between sediment accretion and nutrient availability on clonal growth of wetland macrophytes.

## Conclusion

Our results suggest that the plasticity of clonal growth forms enable *C. brevicuspis* to acclimate to moderate sediment burial, but not to deep sediment burial. Nutrient enrichment can increase the growth and vegetative propagation of *C. brevicuspis* up to moderate sediment burial, but has no effect on clonal growth forms. Nutrient enrichment in sediments could not induce the changes in clonal growth forms and could not facilitate the acclimation of *C. brevicuspis* to heavy sedimentation. In habitats with increasing sedimentation rate, such as many lacustrine wetlands, modification of clonal growth forms might be insufficient to allow *C. brevicuspis* to overcome severe burial stress. Therefore, in such habitats *C. brevicuspis* might be less competitive like burial-tolerant species such as *Phalaris arundinacea* L. (Chen et al., 2017).

## Author Contributions

XC and YL wrote the manuscript and executed the technical assays and statistical analysis. XC and YX designed the experiment and edited the manuscript text. FL, ZH, ZD, and CW contributed to data collection and interpretation. All authors reviewed the manuscript.

## Conflict of Interest Statement

The authors declare that the research was conducted in the absence of any commercial or financial relationships that could be construed as a potential conflict of interest.
